# Regulations Governing Sex Selection in Bangladesh: Implications of Islamic Law and Ethics

**DOI:** 10.1007/s41649-025-00399-5

**Published:** 2026-05-05

**Authors:** Sanwar Siraj, Yousuf Ali, Jahid Siraz Chowdhury, Kamrul Hasan

**Affiliations:** 1 Centre for Medical Ethics and Law, Faculties of Law and Medicine, The University of Hong Kong, Hong Kong; 2 Department of Political Science, Dhaka International University, Dhaka, Bangladesh; 3 Lincoln-Yunus Social Business Center, Lincoln University College, Petaling Jaya, Selangor Malaysia; 4 Medical Ethics and Humanities Unit, Li Ka Shing Faculty of Medicine, The University of Hong Kong, Hong Kong

**Keywords:** Medical assisted technology, Sex selection, Health law, Islamic ethics, Bangladesh

## Abstract

Sex selection for medical purposes is a widely accepted method primarily aimed at preventing the transmission of X-linked genetic disorders to future generations. However, sex selection for socio-cultural or nonmedical reasons—e.g., personal preference for a specific gender or family balancing issues—has been a debated issue in health law, public policy, and ethics in many countries, including Bangladesh. Recently, the Bangladesh High Court ordered the government to ban prenatal sex selection for nonmedical reasons. In response, the Directorate General of Health Services (DGHS) proposed national guidelines for healthcare providers, requiring hospitals, diagnostic centers, and clinics to strictly prevent the disclosure of fetal sex during pregnancy. This paper argues that the guidelines should be updated to include a ban on all forms of sex selection for nonmedical reasons. Islamic law prohibits all types of nonmedical sex selection because they are against the will of Almighty Allah. Muslims believe that Allah alone creates human life and that humans do not have the authority to determine the sex of the fetus. International documents and conventions also oppose sex selection for nonmedical reasons, viewing it as discriminatory, especially in societies with deep-rooted gender biases. Therefore, this paper asserts that a comprehensive legal framework—specifically enacted by the Bangladesh Parliament—is necessary to ban all forms of sex selection for nonmedical reasons. Additionally, legislation alone may not be sufficient, as it might unintentionally allow discriminatory practices to continue elsewhere in society. Instead, a holistic approach is needed—one that includes socio-cultural shifts through education and awareness campaigns promoting gender equality, the empowerment of women and girls, and their economic independence. These strategies are crucial for addressing the root causes of gender-based preferences and building a more equitable society.

## Background

Sex selection refers to attempts by couples to influence or determine the sex of their future child (Serour 2023). Broadly, these practices are either natural—such as timing intercourse or adjusting diet—or medically assisted, including laboratory techniques used before or after conception (Bokek-Cohen and Tarabeih 2020). Medically assisted methods include preconception (sorting X- and Y-bearing sperm), preimplantation (selecting embryos of a specific sex during in vitro fertilization, IVF), and prenatal (determining fetal sex during pregnancy, sometimes leading to selective abortion) (Dondorp et al. 2013).

Modern medical technologies now allow sex selection before conception. In the preconception stage, sperm can be separated into X- and Y-bearing samples using fluorescent staining and laser sorting; this method yields roughly 90% accuracy (Karabinus et al. 2014; Naidu et al. 2025). In the preimplantation stage, embryos created through IVF are genetically screened (PGD) to identify both chromosomal health and sex, allowing only selected embryos to be transferred to the uterus (Zuckerman et al. 2017). This method has success rates exceeding 95% (Zuckerman et al. 2017). Lastly, parental sex selection is carried out after the conception period, which involves techniques such as ultrasonography or genetic testing to determine the genetic makeup and the sex of the fetus. These methods can provide highly accurate results with a high level of precision (Colmant et al. 2013).

The World Health Organization (WHO) reports that sex selection is primarily carried out for medical, nonmedical, and family balancing issues (WHO 2011). Sex selection is typically carried out to prevent the birth of a child with a serious sex-linked genetic disorder. Sex selection is highly effective, and its use for medical purposes is legal in most countries worldwide. Its use for non-medical reasons has been subject to ethical controversy, disapproval, and even condemnation (Serour 2023). While the primary justification to use the medical technologies is to prevent the transmission of sex-linked genetic disorders and to manage health-related risks, they are frequently used for nonmedical reasons—such as personal preference, particularly for sons—and for family balancing, especially when families have one or more children of the opposite sex (WHO 2011). Sex selection for nonmedical reasons often fulfills couples’ gender preferences for a child of a specific sex (Dondorp et al. 2013). It raises serious ethical concerns when medical technology is used to manipulate reproductive materials or when people have a strong preference for a specific gender. Often, this leads to the selective implantation of male embryos or sex-selective abortions. Sex selection for nonmedical reasons can encourage couples to favor one gender over another, usually males. Sometimes, couples choose the sex they prefer before conception. If the fetus is of an unwanted sex, they may decide to terminate the pregnancy.

In many developed societies, where there is little or no discrimination between the sexes, couples typically wish to have sex selection before conception. This understanding may help couples have children of both genders and exercise their reproductive autonomy, provided they receive appropriate counseling (Serour 2023). Conversely, in countries like Bangladesh, where gender discrimination against girls is widespread and son preference is pervasive, there is a tendency to favor male children, and sex selection for this purpose is more common (Whittaker 2011; Nahar 2022). A recent study has shown that couples in countries with patriarchal family structures and a strong preference for sons are more likely to use medical assisted technology for sex selection (Kashyap and Villavicencio 2017). Other studies have found that in societies where son preference is deeply rooted, the failure to bear a son has sometimes led couples to undergo sex-selective abortions (Bumgarner 2007; Hesketh et al. 2011; Abrejo et al. 2009; Cusack 2013). Studies have also revealed that sex selection is often linked to socioeconomic and cultural factors, societal norms, and the availability of medical technologies (Chakravarty et al. 2022; Nanda and Ray 2020; Fasouliotis and Schenker 1999), even though many jurisdictions prohibit sex selection unless it is medically necessary (Chakravarty et al. 2022; Jejeebhoy et al. 2015).

Bangladesh is a Muslim-majority society, with over 90% of the population being Muslims (Siraj et al. 2024; BBS 2022). The daily lives of Bangladeshi Muslims are generally guided by Islamic ethical norms (Siraj 2021). These Islamic religious norms shape societal values and healthcare decisions (Siraj 2023), including sex selection issues. Many Bangladeshi couples frequently undergo sex selection procedures for personal preference or family balancing issues (Farid 2024). However, Bangladesh has no specific laws regulating sex selection issues (Nahar 2022; Farid 2024). Recently, the Directorate General of Health Services (DGHS) issued a national guideline to prohibit hospitals, diagnostic centers, and clinics from disclosing the sex of the fetus during pregnancy (The Daily Star 2020). The goal of this paper is to review the current practices, guidelines, and regulatory frameworks regarding sex selection. It explores Islamic ethical foundations underlying sex selection practices. Additionally, the paper reviews international documents as well as their practical implications and ethical reasoning within Bangladeshi society and religious culture.

## Regulations and Practices of Sex Selection in Bangladesh

The healthcare system in Bangladesh is hierarchically structured from top to bottom. At the top and national level, the Ministry of Health and Family Welfare (MOHFW) prepares national policies and plans for healthcare, and the Directorate General of Health Services (DGHS) functions as the wing of the ministry to implement health programs, services, policies, laws, and guidelines throughout the country. Bangladesh has three types of healthcare service delivery facilities: public, private, and international donor-supported hospitals (Siraj et al. 2024). Public hospitals typically provide healthcare services either free of charge or at subsidized rates (Sarwar 2015). Hospitals in the capital city generally offer specialized care. Divisional hospitals provide tertiary care. District hospitals offer both secondary and primary care, while upazila health complexes (sub-district level) provide basic and primary care. Union health and family welfare centers offer a range of primary care services to rural populations, including family planning, maternal and child health, immunizations, and treatment for general illnesses. Community clinics at the village (ward) level provide essential primary healthcare to rural populations, often acting as the first point of contact for medical care. The vast majority of Bangladeshi people, particularly those from low- and middle-income groups, depend on publicly funded hospitals for medical care and healthcare services, despite the scarcity of necessary facilities and frequent overcrowding (Abdallah et al. 2022; Rahman 2022; Siraj et al. 2021). Private hospitals, including diagnostic centers and clinics, are profit-oriented but are more accessible, and many patients prefer them to avoid long wait times commonly experienced in public hospitals. Hospitals supported by donor agencies are few and mostly concentrated in semi-urban and urban areas. While some donor-funded hospitals offer direct subsidies to marginalized communities, most healthcare services are not entirely free.

Preconception medical care and health facilities, such as fertility treatment, including basic tests for health issues that could affect or influence pregnancy, folic acid supplementation for women planning pregnancies, and family planning services, are available in Bangladesh. The facilities offering selective fertilization by enriched X- or Y-bearing sperm are not currently available. This advanced technology, which employs flow cytometric sorting based on DNA content to separate X- and Y-sperm, is complex and highly specialized. Centers providing IVF facilities in Bangladesh are limited to urban areas with primarily private settings. Couples use IVF care primarily for infertility treatment who experiencing reproductive challenges. Many fertility centers offer a comprehensive range of infertility treatments, including intrauterine insemination, intracytoplasmic sperm injection, and ovulation induction (Nahar 2022). These options aim to address various causes of infertility and enhance the likelihood of successful conception (Farid 2024). Some couples who can afford it also receive such care for family balancing issues. The cost of a basic IVF cycle typically ranges from 300,000 to 350,000 BDT, with PGD adding to the overall expense. It has begun in recent years, and the success rate is still in its early stages. Many couples who can afford to travel to neighboring countries, such as India, Thailand, and Singapore, have access to IVF care.

Prenatal sex selection is a widely used medical procedure in Bangladesh. This medical technology was first introduced in the 1980 s at urban hospitals and subsequently extended to district and sub-district facilities during the 1990s. Ultrasonography or PGD has been commonly used as a diagnostic tool to have sex selection of fetuses, and the cost for this method is not expensive and easily accessible across various income groups (Naved et al. 2024). Many couples—particularly from low- and middle-income families—undergo sex selection procedures, particularly for nonmedical reasons. A national survey found that women expressing son preference were about 1.5 times more likely to use an ultrasound, and over four-fifths of users sought it for sex determination (Nabi 2018). Although abortion is prohibited under sections 312–316 of the Penal Code of 1860 except to save a woman’s life, an estimated 1.2 million induced abortions occurred in 2014, many in unsafe conditions (Guttmacher Institute 2017).

Sex selection has deep roots in Bangladesh’s patrilineal and patriarchal social order. In societies like Bangladesh, there is a strong preference for sons to continue the lineage, provide economic support, and ensure parents’ security in old age (Kilani and Hassan 2002). Men typically serve as primary earners, while women manage households and child-rearing, reinforcing gendered dependence (Siraj et al. 2024). Daughters are generally viewed as temporary members of the parental household until marriage. Only about 2 percent of Bangladeshi women report daughter preference, while poorer households show a stronger preference for sons who can contribute financially (Fuse 2008). The birth of a firstborn son often symbolizes family prestige and stability (Rahm 2020). Under these conditions, reproductive choices are heavily influenced by men, whose authority in household decision-making extends to healthcare and fertility matters (Nasir and Kalla 2006). Such pressures, intertwined with inheritance traditions and social expectations, continue to drive many couples—especially from lower-income groups—to pursue prenatal sex identification and, in some cases, sex-selective abortion (Khan 2023; Das Gupta et al. 2003).

In traditional societies like Bangladesh, the socio-cultural context is such that women are more dependent on men at every stage of their life cycle. Men tend to hold more power within the societal hierarchy and have greater access to economic resources (Nasir and Kalla 2006). Men, therefore, play a dominant role in making decisions within families, including healthcare, while women’s involvement remains limited. The decision about whether a woman will have a son or a daughter is often made by the husband, especially in lower-middle-income families. The desire to have a son to continue the family lineage and preserve heritage remains a core societal value for many Bangladeshis.

As mentioned above, Bangladesh has no specific law regulating sex selection issues (Nahar 2022). Health care centers providing sex selection or infertility care primarily follow international norms and/or self-directing guidelines (Farid 2024). However, the High Court Division of the Supreme Court of Bangladesh issued a rule on 3rd February 2020, directing the government to explain only why it should not be ordered to prohibit the sex detection of fetuses to protect both the unborn babies and pregnant mothers (The Daily Star 2020). The Court also requested that respondents—various government bodies—explain why their failure to formulate a policy guideline to prevent gender-biased sex selection should not be declared illegal. Respondents to this rule included the Secretaries of the Ministries of Health, Women and Children Affairs, and Social Welfare, as well as the Directors General of the Directorate General of Health Services (DGHS), Family Planning, the Department of Women Affairs, and the National Institute of Population Research and Training. Additionally, the petitioner’s lawyer sought an explanation from these respondents as to why they should not be directed to maintain a database of diagnosis reports of unborn babies conducted by registered hospitals, diagnosis centers, and other entities. Subsequently, the DGHS formulated the National Guideline for Prevention of Son Preference and the Risk of Gender-Biased Sex Selection in 2022, which was submitted to the High Court for consideration (Faisal 2024). The guidelines issued six key principles (Islam 2024):

1. No individual, organization, genetic counseling center, laboratory, or clinic—including those equipped with ultrasound, imaging, or other technologies capable of determining fetal sex—shall offer, display, publish, distribute, or communicate in any manner information about facilities for prenatal diagnosis or sex determination.
2. In the context of infertility treatments, no individual or organization—including clinics, laboratories, or centers capable of conducting sex selection on individuals or derived cells—shall provide, display, publish, or communicate information related to facilities for prenatal sex selection.
3. The relevant government ministries shall undertake efforts to raise awareness among stakeholders—including, but not limited to, gynecologists, medical geneticists, pediatricians, registered medical practitioners, community health and family planning workers (such as midwives, community skilled birth attendants, family welfare assistants, sub-assistant community medical officers, family welfare visitors), radiologists, sonologists, imaging specialists, nurses, and technicians—regarding the risks associated with gender-biased sex selection.
4. The aforementioned stakeholders should be informed, among other things, about the Code of Professional Conduct, Etiquette, and Ethics issued by the Bangladesh Medical and Dental Council, which explicitly stipulates that sex determination for social, cultural, or non-medical reasons should not be performed.
5. Any individual, organization, genetic counseling center, laboratory, or clinic—including those equipped with ultrasound machines, imaging devices, scanners, or any other technology capable of determining the sex of the fetus—must systematically collect, document, and preserve records of all screenings performed at their respective facilities.
6. Any individual, organization, genetic counseling center, laboratory, or clinic—including those equipped with ultrasound machines, imaging devices, scanners, or any other relevant technology—capable of determining the sex of a fetus are required to display messages—either digitally or in print (e.g., posters)—that promote gender equality and emphasize the value of girl children.

Adopted in response to the High Court’s directive in 2020, these guidelines prohibit medical professionals and health staff from disclosing fetal sex during pregnancy. While ultrasonography serves various legitimate purposes—such as monitoring fetal development, ensuring maternal and fetal health, and satisfying parental curiosity—its misuse in Bangladesh has primarily led to the induced abortion of female fetuses, driven by socio-economic factors, family preferences, and patriarchal traditions. In February 2024, the High Court Division directed the Bangladesh government to ensure strict adherence to these guidelines across hospitals, clinics, and diagnostic centers, and it has only placed an embargo on prenatal sex detection for nonmedical reasons. This measure is in place to prevent gender-biased sex selection, which often involves the abortion of female fetuses due to son preference. The national guideline only permits sex disclosure for medical reasons, such as in cases of sex-linked genetic disorders or other health risks. This paper addresses a set of ethical questions:

- What about the Islamic ethical position on sex selection?
- How can these national guidelines uphold international commitments and ethical standards—such as the Cairo Convention, Beijing Convention, Convention on the Elimination of All Forms of Discrimination Against Women (CEDAW), the Convention on the Rights of the Child (CRC), the International Covenant on Civil and Political Rights (ICCPR), and the European Convention on Human Rights and Biomedicine (Biomedicine Convention)—regarding sex selection issues?
- How effectively are the national guidelines being enforced, and are these guidelines reflected in hospital, diagnostic, and clinical practices?
- What gaps remain, and what types of regulations could the Bangladeshi government develop to more effectively address all aspects of sex selection issues?

## Islamic Ethical Principles regarding Sex Selection

This section presents Islamic ethical arguments on sex selection issues. Islamic law (Shari’ah) is derived from two primary sources: the Quran, which contains the literal words revealed to the Prophet Muhammad by the Angel Gabriel, and the Sunnah, encompassing the sayings, actions, and practices of the Prophet Muhammad. If there is nothing directly addressed in the primary sources regarding modern issues, Muslim scholars resort to secondary sources such as Ijma (consensus), Qiyas (analogy), and Ijtihad (independent reasoning), as well as subsidiary tools like Istihsan (preferential reasoning), Urf (custom), and Maslahah (public interest) (Sachedina 2009; Atighetchi 2007). Together, these guide ethical decision-making in contemporary medical, healthcare, and research contexts (Chamsi-Pasha and Albar 2019; Ibrahim et al. 2019).

The moral legitimacy of using reproductive medical technology to determine the sex of future offspring remains contested (Eftekhaari et al. 2015). Some argue that couples may have the right to influence offspring sex, while others view such acts as interfering with divine will and the natural order (Benagiano and Bianchi 1999). Human-rights advocates warn that gender preference may reinforce inequality and devalue the opposite sex (Sermon et al. 2007). These debates lead to a central question: what is Islam’s position on sex selection?

Islamic law limits the use of assisted reproductive medical technologies to only lawfully married couples (Farid 2024; Husain 2000). Islamic scholars generally support medical intervention, such as IVF, for infertility treatment (Al-Bar and Chamsi-Pasha 2015). They base their view primarily on the verse “Wealth and progeny are adornments for the life of this world” (Quran 18:46) as a justification for seeking future offspring (IMANA Ethics Committee 2005). Accordingly, fertility treatment such as IVF is permissible when used to overcome infertility within legal marriage. Muslim scholars support sex selection that requires medical necessity, particularly to prevent serious X-linked genetic disorders or hereditary diseases. For example, sperm sorting can be used to select sperm that are less likely to carry certain genetic defects, thereby reducing the risk of transmitting those defects to the embryo. The acceptance of PGD is that it avoids the implantation of defective embryos, thereby eliminating the need for future pregnancy termination (Schenker 2002). An international conference on bioethics in human reproduction research within the Muslim world was held in Cairo from October 10 to December 13, 1991. The conference was attended by approximately 200 participants, including doctors, Muslim scholars, and theological experts. During this gathering, the consortium declared that “sex selection is permissible in cases where a specific sex is predisposed to a serious genetic condition” (Serour et al. 1992). Using these technologies for nonmedical reasons, e.g., personal preference or family balancing, raises ethical objections, as it departs from the principle of accepting the Almighty Allah’s decree in creation.

Scholars who support preconception sex selection for nonmedical reasons, such as in the case of personal preference or family balancing issues, are in the minority among Muslim scholars. Proponents of this view often cite the Quranic verse: “And you do not will except that Allah wills — the Lord of the worlds” (81:29). This verse underscores the concept that all human desires and intentions are ultimately subject to the will of Allah, the Creator and Sustainer of all existence. Supporters argue that since human beings can only act within the confines of Allah's divine will, they should be permitted to pursue choices like sex selection, as long as these choices do not explicitly contravene Islamic prohibitions. Some scholars argue that, since neither the Quran nor the Sunnah explicitly prohibits sex selection, it may be regarded as permissible by default. They base their argument and cite the Prophetic tradition recorded in Sunan al-Tirmidhi (Hadith, 1726): “Whatever is not expressly forbidden is permissible.” On this basis, they contend that personal preference or family harmony can justify the use of such technologies, provided there is no harm or violation of moral principles. From this view, the higher objectives of Islamic law—preserving life, lineage, and family stability—are not inherently threatened by preconception sex selection when intentions are benign.

However, the vast majority of jurists reject this permissive stance. While procedures such as sperm sorting or PGD may technically avoid abortion, they still attempt to alter outcomes that are considered to be within divine decree. They emphasize that intentionally influencing the sex of a child, even before conception, constitutes interference with Allah’s creative will. Scholars cite the following Quranic verse: “He creates what He wills; He bestows female upon whom He wills and male upon whom He wills” (42:49), interpreting it as proof that determining a child’s sex lies solely within God’s authority. Accordingly, deliberate manipulation of fetal sex for preference or convenience is considered impermissible unless medically necessary, such as to prevent X-linked genetic disorders (Athar 2008; Al-Bar and Chamsi-Pasha 2015).

Scholars argue that for couples undergoing IVF who have a history of being carriers for autosomal recessive conditions, embryos can be tested, and those confirmed not to carry the genetic defect can be selected for implantation, which is permissible (Shoaib 2024). The embryos are considered the exclusive property of the married couple and may be transferred to the same wife in subsequent cycles. Preimplantation procedures are permissible in Islamic law because they occur before ensoulment, which is believed to happen at 120 days after fertilization.^1^ This option can be particularly relevant for Muslim families with a history of inherited disorders (Thong et al. 2018). However, scholars prohibit couples from “intentionally” using medical technologies to influence the sex of embryos or fetuses for nonmedical reasons. Preimplantation techniques involve selecting healthy embryos of a desired sex before implantation into the uterus. During this period, while healthy embryos of the “desired” sex are selected for transfer, others may be discarded or stored. Muslim scholars view that couples often tacitly use infertility treatments involving determining the gender of IVF embryos and selectively transferring the “desired” male or female healthy embryos. Such practices should be considered impermissible unless there is a valid medical necessity, as they can be seen as interfering with God’s creation and may lead to unethical social consequences. Sex selection is considered an alteration of the Almighty’s creation—particularly when motivated by nonmedical reasons, such as personal preference or family balancing. Quranic teachings emphasize that the Almighty is the sole bestower of male and female offspring, and the principle often cited by Muslim scholars is that “there is no change in the creation of Allah” (30:30). These ethical perspectives encourage Muslims to oppose preconception methods of sex selection using reproductive technologies for reasons other than medical necessity (Chamsi-Pasha and Al-bar 2019).

A relevant ethical question often cited in Islamic bioethics literature is whether Islamic law permits sex selection in cases of urgent necessity, such as addressing family imbalances—particularly when families lack children of a specific gender, like a son or a daughter. In such situations, scholars argue that prospective couples may seek to determine the sex of their offspring before conception or during the preimplantation stage to ensure the presence of a caregiver, a provider for old age, or an heir to inherit family assets. For example, many scholars consider sex ratio balancing acceptable only in very limited and specific circumstances. One such scenario involves cases where a woman has already given birth to three or four children of the same sex—either all daughters or all sons—and it is deemed in her and her family’s best interest that her subsequent pregnancy results in a child of the opposite sex. This may be particularly relevant if the subsequent pregnancy is intended to be her last, and the family wishes to achieve a more balanced gender ratio within the household (Serour 2008). These methods of sex selection are generally viewed as permissible under conditions of extreme necessity or for purposes of family balancing, which aims to address familial needs rather than personal preferences (Muhsin et al. 2023, 223–232). Islamic scholars support this exceptional approach by referring to the maxim that “extreme necessity (al-dharoorah) makes unlawful things permissible,” thereby permitting certain actions that would otherwise be prohibited under extraordinary circumstances. Scholars cite the Quranic verse, “Allah has not laid upon you any hardship in religion” (22:78), to justify such allowances, emphasizing that Islam recognizes the importance of alleviating hardship and addressing genuine needs. They argue that in these specific contexts—such as correcting gender imbalance for family balancing or health reasons—sex selection does not constitute discrimination or personal preference but rather serves a legitimate purpose aligned with Islamic ethical principles.

However, scholars are clear that this permissibility does not extend to choosing a child's sex solely based on parental preference for esthetic, social, or personal reasons, which is strongly condemned within Islamic law and ethics. Such preferences are considered unjustifiable and contrary to the spirit of fairness and divine guidance. Instead, the focus is on addressing genuine needs or mitigating hardship, ensuring that the practice of sex selection remains within ethical and religious bounds (Serour 2008).

Islamic scholars view prenatal sex selection—the identification of a fetus’s sex during pregnancy for nonmedical reasons—as ethically problematic because it may lead to sex selective abortion if the fetus is not the desired sex. The Islamic Fiqh Council of the Muslim World League issued a fatwa in 2007 prohibiting such practices, warning that they foster gender discrimination, social imbalance, and the devaluation of female children (Chamsi-Pasha and Al-Bar 2015). In Islam, abortion is forbidden except in special circumstances. It may be permitted if continuing the pregnancy endangers the mother’s life or causes serious physical or mental harm, provided it occurs before 120 days of gestation—the stage believed to mark ensoulment (Hosen et al. 2021). Termination is also permissible before that stage if the fetus has severe, incurable anomalies, and at any time if the mother’s life is at risk. Muslim jurists are unanimous in rejecting abortions carried out solely for sex preference. The Quran strongly condemns infanticide: “Kill not your children for fear of want; We shall provide sustenance for them and for you. Verily the killing of them is a great sin” (17:31). Because such procedures discriminate against female fetuses and perpetuate social injustice, many religious authorities uphold fatwas restricting sex-selection technologies to medical necessity only (Bokek-Cohen and Tarabeih 2020; Serour 2008). Many Muslim countries, for example—including all Arab states except Tunisia—prohibit sex-selective termination of pregnancy (Chaabouni-Bouhamed 2008).

## International Documents and Frameworks Governing Sex Selection Issues

This section outlines the regulatory frameworks established by various international organizations, treaties, and conventions concerning sex selection issues. These laws and frameworks can serve as essential references for countries like Bangladesh in developing policies and regulations aimed at preventing sex selection for nonmedical reasons or family balancing issues, particularly practices driven by gender bias (Radicic 2008). In this section, we have also outlined the extent to which the national guidelines issued by the Bangladeshi health authorities align with these international standards and identified existing gaps and proposed a structured policy measure, if necessary, for Bangladesh to effectively address the challenges related to sex selection.

The CRC affirms every child’s right to equality and protection from discrimination. Article 2 prohibits any distinction based on gender or other status. Moeckli (2017) opines that prenatal sex selection that favors one gender directly contravenes these principles by reinforcing stereotypes that devalue female children and violate their rights to equal treatment and protection. Similarly, the ICCPR obliges states to uphold equality before the law. Article 26 of this Convention guarantees equal protection and freedom from discrimination. Moeckli (2017) argues that allowing sex selection for nonmedical reasons undermines these rights by perpetuating gender bias and systemic inequality, contrary to the spirit of the ICCPR.

The Biomedicine Convention explicitly prohibits using assisted-reproduction techniques to choose a child’s sex except to avoid a serious hereditary, sex-linked disease (Article 14). This restriction aims to prevent misuse of reproductive technologies and uphold ethical and anti-discriminatory standards. Although the Convention does not regulate sex-selective abortion, its prohibition of pre-conception and pre-implantation sex determination aligns with Islamic law and offers guidance for patrilineal societies such as Bangladesh, where gender bias remains widespread.

The Program of Action adopted by 184 UN Member States at the 1994 Cairo Conference on Population and Development represented a significant milestone by explicitly addressing issues such as prenatal sex selection (sex-selective abortion) and female infanticide. Article 4(1) of this document emphasizes the need to eliminate all forms of discrimination against the girl child and tackle the root causes of son preference, which often lead to harmful and unethical practices such as female infanticide and prenatal sex selection. The Platform for Action, adopted by 187 UN Member States at the 1995 United Nations Fourth World Conference on Women, that held in Beijing, further reiterates the importance of measures to combat sex-selective abortion and infanticide.

The UN Convention on the Elimination of All Forms of Discrimination Against Women (CEDAW), adopted in 1979, defines discrimination as any distinction or restriction based on sex that undermines women’s rights (Article 1). Article 5 of the CEDAW obliges States Parties to modify social and cultural patterns that reinforce gender stereotypes, while Article 16(1) affirms equal rights for men and women to decide freely and responsibly on the number and spacing of their children. Consistent with the Cairo and Beijing Declarations, however, this provision does not extend to choosing the sex of one’s children (Toebes 2008).

International human-rights standards and treaty-monitoring bodies consistently oppose sex selection. When aimed at producing sons, it is viewed as discriminatory and, in some contexts, a form of violence against women. Toebes (2008) argues that reproductive choice does not include the right to select a child’s sex—an especially important limitation in societies with entrenched gender bias. These principles apply equally to prenatal procedures such as sex-selective abortion, which violate the obligation of states to protect women and girls from practices perpetuating inequality.

Scholars often raise a question about whether couples have a right to know the sex of their unborn child (Toebes 2008). Toebes (2008) argues that CEDAW’s Article 10(h) guarantees women equal access to education and family-planning information but was never intended to create a right to fetal sex disclosure. As a signatory to CEDAW and the Convention on the Rights of the Child (CRC), Bangladesh is bound to uphold gender equality and prohibit discrimination. Nationally, the Penal Code of 1860 (Sections 312–316) maintains a ban on sex-selective abortion except when performed in good faith to save the mother’s life (Guttmacher Institute 2017).

International documents oppose all forms of social discrimination, and sex selection for nonmedical reasons often threatens societies where gender preference is widespread. These international standards are reflected in court rulings and national guidelines, but the current guidelines remain inadequate in addressing all forms of sex selection. For example, the national guidelines only instruct hospitals, clinics, and diagnostic centers not to disclose the sex of the fetus. This gap could be addressed by including issues related to preconception and preimplantation sex selection. However, another limitation is that these guidelines are not legally binding. While they can serve as a foundation, effectively addressing gender discrimination will likely require the enactment of comprehensive legal frameworks by the parliament.

## Regulatory Proposals and Ethical Framework Governing Sex Selection in Bangladesh

Since the formulation of the guidelines, the DGHS has taken several proactive steps to ensure their effective enforcement. The DGHS has mandated that all diagnostic centers, clinics, and hospitals equipped with ultrasound or imaging facilities must register with the DGHS, facilitating closer monitoring and oversight of facilities performing prenatal testing. This initiative aims to scrutinize and regulate diagnostic centers, hospitals, and clinics involved in prenatal sex determination, thereby helping to prevent gender-biased sex selection and selective abortion. In addition, the DGHS has launched training programs for health professionals, including gynecologists, radiologists, nurses, and technicians, focusing on ethical standards and the potential risks associated with prenatal sex selection. Several healthcare staff members have already received training under this initiative. The DGHS also emphasizes the importance of promoting gender equality by instructing diagnostic centers, hospitals, and clinics to display messages that highlight the value of girl children and advocate for gender equity. Some centers and clinics have already implemented this practice in accordance with the guidelines, signaling a positive step toward changing societal perceptions and reducing gender bias.

Although the guidelines set clear standards, they are not fully followed in hospitals, diagnostic centers, and clinics. For example, many of these places still perform nonmedical sex selection. Health staff are not enough health staff to monitor all activities effectively. While the guidelines have increased public awareness and some facilities have adopted internal policies, enforcement is inconsistent. There is no dedicated health department to systematically collect and analyze data on prenatal sex selection reports. This makes it difficult to detect violations and enforce the rules.

Another problem is the growth of unregistered diagnostics and clinics, especially in rural areas. Many of these clinics lack proper licensing and oversight, making law enforcement difficult (Farid 2024). Limited financial resources also hinder monitoring. Healthcare spending is very low—less than 2.5% of the per capita Gross Domestic Product (GDP)—mostly going to primary healthcare and family planning (World Bank 2022). In comparison, countries like the Maldives spend about 9.65%, and Nepal spends around 6.66% of their GDP on healthcare (World Bank 2022).

Private ultrasound centers and diagnostic clinics are mainly profit driven. This profit motive can encourage staff to ignore legal restrictions, risking violations. The weak rule of law and widespread corruption in the healthcare sector also make enforcement difficult (Naher et al. 2020).

Finally, there is a significant gap in comprehensive laws and regulations necessary to effectively enforce existing measures. Currently, these are only guidelines, not statutory law, that do not constitute legally binding principles that prohibit diagnostic centers and clinics from disclosing the sex of fetuses to couples. The BMDC’s Code of Professional Conduct, Etiquette, and Ethics also prohibits sex selection for social, cultural, or other nonmedical reasons, but the BMDC is not functioning due to the legal frameworks. We should recognize that the enactment of a law and related regulations would create a binding legal framework, compelling stakeholders to adhere to restrictions and reducing the likelihood of violations. This highlights the urgent need for parliamentary legislation, complemented by an ethical framework and the development of robust enforcement mechanisms, to effectively combat gender-biased sex selection practices and promote gender equality.

We recognize that the DGHS guideline represents an initial step toward a just society where individuals are not discriminated against based on gender. We call for updating the existing guidelines to include all forms of sex selection and to ensure proper implementation across the country. We recognize that ongoing progress requires combining legal measures with broader social efforts to address deep-rooted gender bias. Regulating sex selection for nonmedical purposes is essential, as such practices are banned or heavily restricted in most countries. Many countries with similar socioeconomic conditions, such as India, have enacted prohibitions on prenatal sex selection (Bhatia 2021; Kaur and Kapoor 2021).

Therefore, parliamentary legislation is crucial to establishing a comprehensive framework ensuring the ethical use of reproductive technologies across all hospitals, diagnostic centers, and clinics in Bangladesh. This law should clearly define eligibility criteria for health care procedures, balancing individual rights with societal interests (Thompson et al. 2006). A robust legal structure serves not only as a professional guide for healthcare providers but also as a foundation for ethical conduct and public accountability (Willcox 1964). Embedding ethical principles within legislation may help prevent misuse of reproductive technologies, promote gender equity, and ensure that all individuals—regardless of gender or socioeconomic status—receive fair treatment.

So, enacting a comprehensive parliamentary legal framework and ethical guidelines is necessary to explicitly regulate all forms of sex selection. To align with Islamic ethics and international documents, Bangladesh should enact laws that explicitly ban all forms of nonmedical sex selection practices. Current guidelines focus only on prenatal sex selection methods, but this framework should also cover preconception and preimplantation procedures to ensure ethical practice. The laws and policies should include stipulations related to the ban of all forms of sex selection techniques for nonmedical reasons to prevent gender-biased outcomes and reinforce equality.

Limited exceptions may be permitted in extraordinary medical cases, such as families with a history of sex-linked genetic disorders or personal preferences, or those with multiple children of the same gender seeking genuine family balance. Such cases must undergo rigorous ethical and medical review. Strict oversight is crucial to prevent misuse, especially in a culture where son preference remains strong. Health facilities should be required to document such procedures to ensure compliance with ethical standards and to discourage discrimination against female children. To ensure oversight, we recommend creating a dedicated regulatory wing within the DGHS to evaluate applications, license practitioners and clinics, and monitor compliance. The law should include strong sanctions for violations—such as fines, license revocation, and legal action—to uphold professional accountability and deter unethical practices.

Many have concerns that the law alone may be insufficient, given the deeply rooted societal norms and cultural preferences for male offspring that are widespread in many societies (Purewal 2010; Goodkind 1999). Social scientists refer, for example, to the Indian Pre-Conception and Pre-Natal Diagnostic Techniques Act of 1994 (amended in 2003), which prohibits gender-biased sex selection; but the practice remains widespread and often conducted covertly (Chakravarty et al. 2022). Similarly, in Iran, regulations prohibit the use of ultrasound for nonmedical sex selection purposes, such as sex selective abortion. However, enforcement remains inconsistent, and socio-cultural factors, like a preference for sons, may motivate couples to resort to clandestine procedures to achieve their preferences (Haseli et al. 2024).

We recognize that while legal measures are vital, broader societal change is imperative. Chakravarty et al. (2022) emphasize that fostering socio-cultural shifts to value women is essential for safeguarding against issues related to nonmedical sex selection. Healthcare providers and policymakers must receive training that incorporates religious and moral reasoning into medical protocols. Considering that approximately 59 percent of the population resides in rural areas (World Bank 2024), outreach efforts through mosques, community leaders, and mass media are vital to raise awareness. Collaborating with Islamic scholars and jurists to issue public fatwas and conduct educational campaigns can provide religious legitimacy to legislation, promote acceptance of Allah’s divine will, discourage gender bias, and reaffirm the equal value of female children in accordance with Islamic teachings. Social scientists also underscore the need for multidimensional strategies combining legal reform, education, and economic empowerment (Kabeer 2003). Bangladesh has already reformed several laws to reduce gender discrimination, including the Children Act 2013, Child Marriage Restraint Act 2017, and Dowry Prohibition Act 2018.

## Conclusion

The guideline proposed by the DGHS only addresses prenatal sex selection. Islamic ethical principles prohibit any form of sex selection for socio-cultural or nonmedical reasons, such as personal preference or family balancing. The guideline should be updated to include provisions for other forms of sex selection, such as preconception and preimplantation procedures. Sex selection for nonmedical or family balancing issues should be prohibited because it perpetuates gender discrimination, especially in Bangladesh, where son preference is widespread. Although Islamic scholars conditionally permit exceptions in cases of dire necessity—such as when a woman has had multiple daughters and it is her last pregnancy—using techniques like sperm sorting or PGD to ensure a son may be approved to fulfill family obligations and reduce health risks (Schenker 2002). International documents and conventions explicitly condemn all forms of discrimination.

This paper concludes that the current guideline may not effectively prevent discriminatory practices; therefore, enacting a comprehensive legal framework through the Bangladesh parliament is necessary to protect vulnerable women and girl children. The government should also promote societal and cultural reforms to foster gender equality through educational programs and public awareness campaigns. The government should ensure the effective implementation of reforms across the country. Nationwide initiatives should focus on educating the public—especially in rural areas—about the harms of gender bias and the importance of legal restrictions. Education programs should include learning initiatives that value women, while economic reforms should expand women’s access to employment and reduce dependence on male offspring. Together with community engagement and public awareness, these measures can build a more equitable society that values all children equally. Addressing root causes such as gender inequality and poverty is essential for long-term change. The paper suggests that the Bangladesh government initiate multisectoral collaboration involving government agencies, NGOs, civil society, international stakeholders, community leaders, jurists, and religious scholars to safeguard vulnerable children and pregnant women, thereby contributing to the development of a just society.

## Footnotes

1 Many may raise a question: When does an embryo or fetus become a person? The answer to this question is that an embryo or fetus is generally regarded as a human being after the process of ‘ensoulment,’ in which the Islamic laws prescribe that occurs around 120 days following conception (see, Jaser and Ahaddour 2025). If diagnostic tests confirm that a fetus is afflicted with an untreatable, life-threatening disease or carries a severe disability, therapeutic abortion is considered permissible and lawful, provided that the pregnancy is terminated before the soul is breathed into the fetus, i.e., within 120 days of gestation (see, el-Hashemite 1997). This 120-day period is based on a Sunnah that outlines the stages of fetal development: during the first 40 days, the embryo is called a sperm drop (*nutfah*); in the next 40 days, it becomes a clinging clot (*alaqah*), and during the final 40 days, it develops into a lump of flesh (*mudghah*). After these three stages, an angel is sent to breathe the soul (*ruh*) into the fetus. Once ensoulment occurs, the fetus is considered a human being with the right to life, and abortion is strictly forbidden except in cases where the mother’s life is in danger.
